# Can Vitamin D Levels Influence Bone Metabolism and Osseointegration of Dental Implants? An Umbrella Review

**DOI:** 10.3390/healthcare12181867

**Published:** 2024-09-17

**Authors:** Eduardo Tallon, José Paulo Macedo, Ana Faria, José Maria Tallon, Marta Pinto, Jorge Pereira

**Affiliations:** 1 Faculty of Health Sciences, Fernando Pessoa University, 4249-004 Oporto, Portugal; tallon.eduardo@clinicadrtallon.com; 2 Department of Oral Medicine, Surgery and Implantology, Faculty of Health Sciences, Fernando Pessoa University, FP-I3ID, 4249-004 Oporto, Portugal; jmacedo@ufp.edu.pt; 3 Ana Faria Dental Clinic, 15220 Bertamiráns, Spain; anafaria.medicadentista@gmail.com; 4 Dr. Tallon Clinic, 1700-094 Lisboa, Portugal; josemariatallon@clinicadrtallon.com; 5 School of Medicine and Biomedical Sciences, Fernando Pessoa University, 4249-004 Oporto, Portugal; marta.pinto@clinicadrtallon.com

**Keywords:** bone metabolism, dental implants, dentistry, osseointegration

## Abstract

Introduction: Due to the large amount of scientific evidence on the subject and the limitations and incongruities in previous reviews, the primary aim of this umbrella review is to gather all the information regarding the importance of vitamin D levels in the osseointegration of dental implants. Methods: The literature search was performed in PubMed, Web of Science, CINAHL Plus, Cochrane Library, and Academic Search Complete throughout the search expression [“vitamin D” AND (“dental implant” OR “dental implants”)]. Results: The initial search yielded 351 results, but at the end of the process, only five systematic reviews were selected. Conclusions: Vitamin D seems to have a positive effect on the osseointegration of dental implants and on the reduction of dental implant failures; however, it is recommended that future studies take into account the limitations mentioned in this study in order to increase the validity and quality of scientific evidence on the subject.

## 1. Introduction

### 1.1. Vitamin D

Vitamin D is a fat-soluble vitamin, and its name refers to both vitamin D2 (ergocalciferol) and vitamin D3 (cholecalciferol). Vitamin D2 comes from the ultraviolet-B irradiation of ergosterol, a steroid found in some plants and, mainly, fungi (vegetable origin), and vitamin D3 is synthesized through ultraviolet irradiation in the skin of animals and humans (animal origin) [1]. Furthermore, foods like egg yolks, fatty fish, and fish liver oil are rich in vitamin D3 [2]; however, nutrition is usually only a minor source of it [3].

Both function as prohormones and are used identically by the body. They are transformed through two enzymatic hydroxylation reactions, in the liver and in the proximal renal tubule [4], into their most active form—1,25-dihydroxyvitamin D2 or D3 [5].

In addition to other functions related to other systems, vitamin D regulates calcium phosphate homeostasis and bone mineral metabolism [6], stimulating the production of osteoblastic bone matrix and optimizing bone remodeling [7]. It increases intestinal calcium absorption, leading to a reduction in parathyroid hormone secretion and less systemic bone resorption, with a possible inhibition of osteoclastogenesis [8]. It is well recognized that prolonged vitamin D deficiency is detrimental to the skeleton and may lead to bone loss [9]. For example, a correlation has been found between low levels of vitamin D and the presence of rheumatic diseases, such as osteoarthritis of the temporomandibular joint, which is associated with severe pain [10].

In this way, vitamin D is associated with cancer, heart disease, high blood pressure, diabetes, arthritis, and others but is mainly crucial for musculoskeletal health, namely for its repair and regeneration. Agoncillo et al., (2023) [11] demonstrated that vitamin D supplementation improves muscle strength after exercise-induced muscle damage [11] and Cochet et al., (2023) [12] suggest that its combination with whey proteins or BCAA can be effective in increasing muscle mass, muscle strength, or performance, even in sarcopenic patients [12]. Furthermore, low levels of vitamin D seem to negatively influence the quality of life. Increased respiratory parameters were observed, which altered SpO_2_ parameters and sleep architecture differences in hypertensive individuals with obstructive sleep apnea, with a reduction in the duration of REM sleep, an increase in wakefulness after sleep onset, and a decrease in sleep efficacy [13].

Despite existing knowledge regarding the importance of vitamin D, there is still a high inconsistency in the definition of vitamin D values. The US Institute of Medicine considers that people with a serum value < 12 ng/mL (30 nmol/L) are at risk of vitamin D deficiency, considers serum levels between 12 and 20 ng/mL as corresponding to a risk of insufficiency, and levels above (≥) 20 as being sufficient [14,15]. The Endocrine Society, together with the European Calcified Tissues Society, International Osteoporosis Foundation, or American Geriatrics Society, established the threshold of deficiency at ≤20 ng/mL (50 nmol/L) and designates levels ≤ 10 as severe deficiency [15,16,17,18]. The Endocrine Society determines that only levels ≥ 30 ng/mL are to be considered normal, designating levels between 21 and 29 ng/mL as insufficient [16].

In Portugal, the estimated national prevalence of vitamin D ≤ 10 ng/mL and <20 ng/mL is 21.2% and 45.4%, respectively. Only 33.4% of the adult population presents values ≥ 20 ng/mL, and no more than 3.6% present values ≥ 30 ng/mL [15]. Furthermore, according to a multicenter study published in 2021, of 491 Portuguese patients hospitalized with a confirmed positive COVID-19 test, 431 (87.7%) patients had deficient/insufficient levels of vitamin D. Additionally, the proportion of patients with deficient levels of vitamin D was higher in the group that died (76%) when compared with the groups of patients with moderate and severe disease [19].

**Figure 1:**
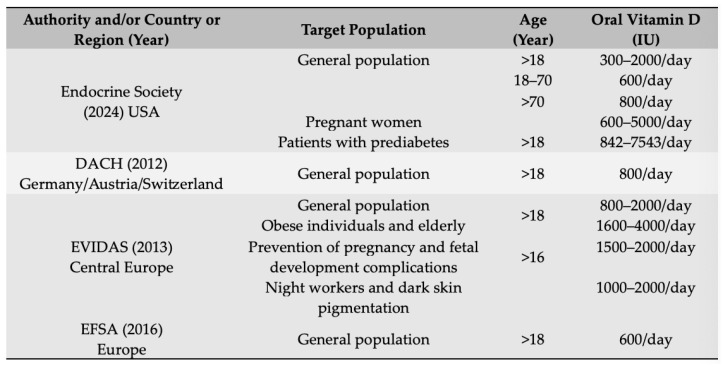
Recommendations for the prevention of vitamin D deficiency in adults.

**Figure 2:**
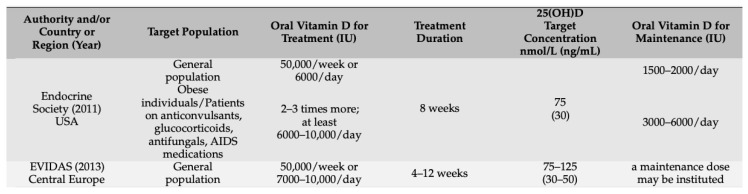
Recommendations for treatment of vitamin D deficiency in adults.

Figure 1 and Figure 2 provide an overview of guideline recommendations, with a focus on Central and Eastern European countries, for prevention and treatment of vitamin D deficiency, respectively, put together by Pludowski et al., (2022) [20].

**Figure 3:**
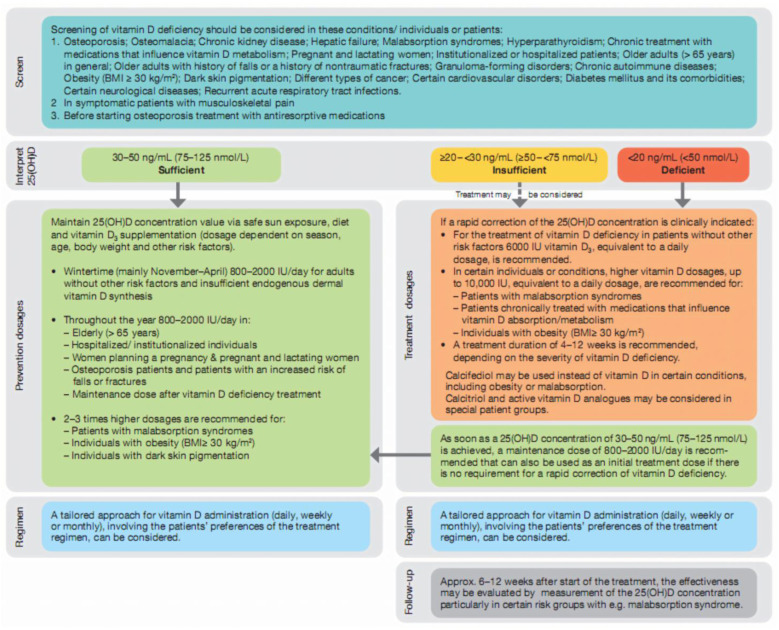
Algorithm for Vitamin D deficiency screening and treatment. Adapted from Pludowsky et al., (2022) [20].

Based on all guideline recommendations and on Pludowski et al., (2022) [20], consensus statements, an algorithm for the prevention and treatment of vitamin D deficiency was developed (Figure 3).

### 1.2. Bone Metabolism

Bone formation occurs through the processes of endochondral ossification and intramembranous ossification [21]. During the first process, the mesenchymal cells differentiate into chondrocytes—chondrogenesis—responsible for the formation of the cartilage growth plate that is gradually replaced by bone [22]. Most of the bones of the human skeleton (long, short, and irregular bones) are formed through this process [23]. The remaining bones are formed by intramembranous ossification. In this process, mesenchymal stem cells differentiate directly into osteoblasts—osteoblastogenesis [24].

In order to preserve the strength and integrity of the bone, it must be constantly replaced. Bone formation by osteoblasts produces organic bone matrix, and bone resorption by osteoclasts dissolves bone mineral and extracellular matrix. In this way, osteogenesis and angiogenesis are two closely associated processes that are involved in bone growth, remodeling, and repair [25].

The success of the osseointegration processes largely determines the perceived bone, and the state of bone remodeling plays a significant role in maintaining optimal quantitative and qualitative characteristics of the jaw bones [26].

To date, the gold standard in the evaluation of bone microarchitectonics is histomorphometric study [27].

### 1.3. Dental Implants

The dental implant is made of alloplastic materials and is implanted in the oral tissues below the mucosa and/or periosteum and/or within the bone, with the aim of providing retention and support for a fixed or removable dental prosthesis [28].

Currently, therapy with dental implants is a treatment option for total or partial edentulism [29,30]. Its performance is indicated in partially edentulous patients who have intermediate gaps or free end edentulism, when a patient is not satisfied with the conventional, unstable, non-retentive full denture, or in situations where it is intended to preserve the existing removable partial dentures [31].

It has advantages over conventional fixed partial dentures. It has a high success rate (over 97%), a low risk of developing caries and endodontic problems of adjacent teeth, better maintenance of bone in the edentulous site, and less sensitivity of adjacent teeth [28].

Over an observation period of 10 years, a survival rate of 85–95% can be estimated, and in 5% the absence of primary implant integration results in implant failure [32].

Dental implant failure (DIF) can occur early—associated with impaired bone healing—or late—after a latency of six months [33]. Early failure is due solely to biological complications that usually involve soft and hard tissue resorption [34].

There are many risk factors that can be associated with DIF, such as iatrogenic, material-associated, patient-related, and systemic factors [32].

Mainly in the last five years, the existing literature on the influence of vitamin D on the osseointegration of dental implants has been increasing; however, there are still studies that argue that there is not enough evidence to establish a correlation or causal relationship between the two factors [35].

Considering the existence of inconsistent information and the increase in the publication of systematic reviews (SRs) on the subject, this document aims to summarize all the information regarding the importance of vitamin D levels in bone metabolism and, consequently, in the osseointegration of dental implants, gathered by SRs, identify the most common and significant failures found in them, and present the respective conclusions and future suggestions in order to guide and establish a new starting point in the development of future studies.

## 2. Materials and Methods

### 2.1. Study Design

This document consists of an umbrella review that is a review and a qualitative analysis of all the information documented only in SRs regarding the influence of vitamin D levels on bone metabolism and consequent success of osseointegration of dental implants.

This umbrella review was carried out from January to April 2023, in accordance with the Preferred Reporting Items for Systematic Reviews and Meta-Analyses (PRISMA) guidelines [36].

Before the start of the review, its protocol was developed and registered in the Open Science Framework (DOI: https://doi.org/10.17605/OSF.IO/EQVGD).

### 2.2. Search Strategy

The existing literature search was performed in five databases: PubMed, Web of Science, CINAHL Plus, Cochrane Library, and Academic Search Complete.

The entire search strategy was developed by two researchers independently, and the search expression used was [“vitamin D” AND (“dental implant” OR “dental implants”)]. All terms used in the search expression correspond to Medical Subject Headings.

The “advanced search” method was selected, and the fields defined in the search were “All fields” in PubMed and Web of Science and “Full text” in the Cochrane Library, CINAHL Plus, and Academic Search Complete.

No limitation was established on the year of publication of the studies.

### 2.3. Eligibility Criteria

As inclusion criteria, the following were defined: (i) SR or meta-analysis (MA); (ii) SR or MA of studies that assess the influence of vitamin D on bone metabolism and, consequently, on the osseointegration of dental implants; (iii) SR or MA of studies performed in humans or animals; and (iv) SR or MA published in Portuguese, English, or Spanish.

As exclusion criteria, the following were defined: (i) SR or MA of studies that only include humans or animals with pathology; (ii) abstracts and commentaries; (iii) irrelevant outcomes that do not demonstrate an association between vitamin D levels and bone metabolism; and (iv) duplicated or incomplete data, which does not allow for a clear and complete understanding of all the data presented, making it impossible to reference them.

### 2.4. Study Selection

The study selection was carried out by two researchers independently.

After searching all published studies corresponding to the established keywords, all duplicates were eliminated.

In the next phase, the remaining studies were analyzed by reading the title and abstract, and most were eliminated.

Subsequently, the full texts of the SR selected and not eliminated in the previous phase were obtained and analyzed through their complete reading, always considering the established eligibility criteria.

At the end of the article selection process, an analysis of the lists of bibliographic references of the SR included so far was made in order to identify any additional SR that met the eligibility criteria.

This first phase was carried out in January 2023, and in March of the same year, the entire process was repeated in order to ensure that the umbrella review encompasses all published literature until its completion.

In each of these phases, meetings were scheduled to discuss the process and reach an agreement in situations of doubt and/or disagreement. In these situations, the suggestions of the two researchers responsible for the study selection were always shared, duly justified, in order to reach a consensus. In cases where consensus was not reached, analysis by a third researcher was requested.

## 3. Methodological Quality Assessment

The evaluation of the methodological quality of the included SRs was carried out by two researchers independently, and the Joanna Briggs Institute (JBI) instrument for SR was used [37,38].

The choice of instrument considered the fact that the SR included other study designs in addition to randomized controlled trials (RCTs).

The purpose of this critical appraisal is to assess the methodological quality of a study and to determine the extent to which a study has addressed the possibility of bias in its design, conduct, and analysis. This tool produces decision-making that considers the feasibility, appropriateness, meaningfulness, and effectiveness of healthcare practice. There are 11 questions to guide the appraisal of the SR or MA, and each must be answered as “yes”, “no”, or “unclear”. “Not applicable” is also provided as an option and may be appropriate in rare instances [37].

The 11 questions are as follows: (1) “Is the review question clearly and explicitly stated?”; (2) “Were the inclusion criteria appropriate for the review question?”; (3) “Was the search strategy appropriate?”; (4) “Were the sources and resources used to search for studies adequate?”; (5) “Were the criteria for appraising studies appropriate?”; (6) “Was critical appraisal conducted by two or more reviewers independently?”; (7) “Were there methods to minimize errors in data extraction?”; (8) “Were the methods used to combine studies appropriate?”; (9) “Was the likelihood of publication bias assessed?”; (10) “Were recommendations for policy and/or practice supported by the reported data?”; and (11) “Were the specific directives for new research appropriate?”.

Meetings were scheduled to discuss the process and reach an agreement in situations of doubt and/or disagreement. As in the study selection phase, to resolve any disagreement in the methodological quality assessment, the suggestions of the two researchers responsible for this phase were always shared, duly justified, in order to reach a consensus. In cases where consensus was not reached, analysis by a third researcher was requested. Inter-rater reliability was not measured.

## 4. Data Extraction and Analysis

This step was also carried out by two researchers.

In a first phase, all main data were identified and placed in a table divided by author(s) and year of the SR, aims of the study, research strategy used, design and number of primary studies included (by the five included SRs), number and characteristics of the sample of primary studies, risk(s) of bias, methodological quality, and authors’ conclusions (Table 1).

Subsequently, whenever possible, the remaining data were analyzed and compared throughout the text.

Data extraction was performed without the aid of any software or instrument. Based on an extensive reading of each study, a summary was made with the most important data from each, organized by points, and finally, these main data were compared manually by the researchers.

## 5. Results

### 5.1. Study Selection

The initial research, through the five databases, yielded 351 results, but after removing the duplicates (51) it boiled down to 300 different studies.

In the second stage, after reading the titles and abstracts, most of the studies were eliminated (292).

In the third stage, only one study was eliminated due to the impossibility of access.

In the fourth stage, through the full reading of the remaining studies, three more were eliminated (Table 2).

In the last phase of the study selection process, no study was eliminated. Thus, five SRs were included in this umbrella review.

**Figure 4:**
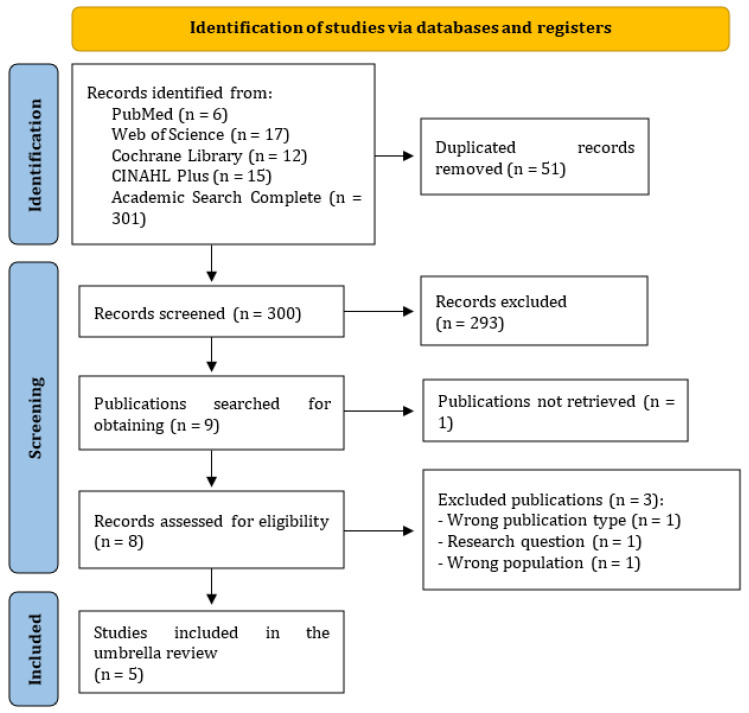
Literature selection process, according to the PRISMA statement. Adapted from Page et al., (2020) [36].

Figure 4 represents the entire literature selection process [36].

After analyzing all the bibliographic references of the five included SRs, no additional SR was found. The SRs included in this umbrella review were published between 2021 and 2022 [39,40,41,42,43].

### 5.2. Methodological Quality

The final score of the SRs included varied between 4 [40] and 9 [43], corresponding to low and high methodological quality, respectively. The other authors had moderate—6 [39] and high—7 [42] and 8 [41] methodological quality (Table 1 and Table 3).

The most frequently satisfied critical items were items 4, 8, 10, and 11, and the least frequently satisfied were items 6 and 7. Item 6 can be related to “lack of a risk of bias assessment” bias, and item 7 can be related to “selective outcome reporting” bias. Item 9 is not applied in any of the SRs since none performed a MA.

### 5.3. Characteristics of the Included Studies

As seen in the eligibility criteria and in the previous table, SRs that analyzed studies with any type of study design were included, due to the small amount of RCTs published on the subject, as perceived in the preliminary research.

The sum of primary studies included in the five SRs corresponds to 51 studies, of which 22 are duplicates. Of the remaining 29, only eight primary studies, published between 2014 and 2021, met the eligibility criteria and therefore moved on to the qualitative analysis phase (Table 4). Makke (2022) [40] and Bazal-Bonelli et al., (2022) [43] included three of them; Hakim et al., (2021) [39] and Werny et al., (2022) [42] included four of them; and Alsulaimani et al., (2022) [41] included five of the eight studies.

Hakim et al., (2021) [39] and Bazal-Bonelli et al., (2022) [43] included two cross-sectional studies. Mangano et al., (2016) [48], Mangano et al., (2018) [50], Hakim et al., (2021) [39], Makke (2022) [40], Alsulaimani et al., (2022) [41], and Werny et al., (2022) [42], included one case report. Fretwurst et al., (2016) [49] and Alsulaimani et al., (2022) [41] included two reviews. Choukroun et al., (2014) [8], Nastri et al., (2020) [51], Hakim et al., (2021) [39], and Bazal-Bonelli et al., (2022) [43] included two RCTs. Garg P et al., (2020) [52], Kwiatek et al., (2021) [53], Hakim et al., (2021) [39], Werny et al., (2022) [42], and Salomó-Coll et al., (2016) [47], included two animal studies.

Through this analysis, it was noticed that none of the SRs included all the existing primary studies in the literature. All of them included studies with main objectives not corresponding to our own [54,55,56]; studies whose implants were placed in other parts of the body, such as in the tibia [57,58,59,60,61,62,63]; femur [24,64,65]; or abdominal region [66]; studies only including subjects with pathology, such as diabetes mellitus [24,58]; chronic kidney disease [65]; or renal failure and secondary hyperparathyroidism [67]; studies that evaluate another variable in addition to vitamin D, not allowing its individual and isolated analysis [35]; or even inaccessible studies [68,69,70].

Furthermore, Werny et al., (2022) [42], in a SR, included the largest number of primary studies—18 studies—and those that included the least amount—four studies—is the most recent SR [43]. Clearly, methodological errors occurred in the latter since the objective of a SR is to include all existing studies about one subject in the literature.

Considering all the flaws identified and mentioned, it is not possible to guarantee that these five SRs collected and analyzed all existing primary studies on the subject. In this way, the need to develop an umbrella review that gathered as much data as possible regarding the importance of vitamin D levels in osseointegration and the success of dental implants and presented all the limitations of existing SRs was reinforced.

Even so, clear homogeneity was observed in the rest of the SRs’ methodologies. They all used the keywords “vitamin D” and “Dental Implant” or “Implant”, but most also used “vitamin D deficiency” and “Implant Failure”.

The most frequently used databases were PubMed, Scopus, Cochrane Library, Web of Science, and Google Scholar.

Regarding the inclusion criteria, the most common were studies evaluating the effect of vitamin D deficiency on dental implants and studies evaluating the effects of vitamin D serum levels on dental implants osseointegration or failure. In addition to these, some SRs still established as inclusion criteria the intended study designs and studies publish in English. The most frequently established exclusion criteria were irrelevant topics, incomplete data, and duplicated studies. Some SRs still excluded in vitro and animal studies, letters to the editor, historic reviews, and commentaries.

Only one of the SRs included specific characteristics of the sample in the eligibility criteria. Although it was not very specific, Alsulaimani et al., (2022) [41] included male-female human adult patients between 18 and 65 years of age.

All SRs were conducted according to the guidelines of the PRISMA statement.

Only two SRs were selected as eligibility criteria: a specific experimental (EG) and control group (CG). Alsulaimani et al., (2022) [41] defined EG as patients with low serum vitamin D level, and CG as patients with normal serum vitamin D level, and Werny et al., (2022) [42] defined EG as “the effect of vitamin D deficiency/supplementation on osseointegration” and CG as “osseointegration with adequate vitamin D serum level”. The remaining SRs only defined the eligibility criteria in a general way, as indicated above; however, all of them evaluated either the effectiveness of optimal levels of vitamin D or the implication of a vitamin D deficit in dental implants, such as indicated in the main objectives of the SR and this umbrella review.

Four of the five SRs defined “success of dental implants” and thus, as outcome measures, “survival rate and marginal bone loss of placed implants” [43], “implant survival” [39], “dental implant failure; peri-implant marginal bone loss; biological or mechanical complications reported at the implant or patient-level”, and [41] “bone-to-implant contact (BIC); new bone formation and/or implant resistance; bone volume/tissue volume around the implants with and without vitamin D deficiency/supplementation” [42]. Makke (2022) [40] was the only study that did not mention outcome measures.

According to Bazal-Bonelli et al., (2022) [43], Alsulaimani et al., (2022) [41], and Makke (2022) [40], a serum vitamin D level of <10 ng/mL is considered deficient or inadequate, a range of 10–30 ng/mL is considered insufficient, and >30 ng/mL is considered a sufficient/optimal serum level. According to Hakim et al., (2021) [39], a 25(OH)D or 25-hydroxyvitamin D of less than 10 ng/mL is considered severe deficiency, a level of 10–24 ng/mL is considered deficiency, and a level of 25–80 ng/mL is considered normal. Werny et al., (2022) [42] did not mention vitamin D reference levels.

## 6. Outcome Measure Analysis

### 6.1. Vitamin D Deficiency and Dental Implant Failure

According to Hakim et al., (2021) [39], 6 of the 12 investigated studies found a significant association between vitamin D deficiency and DIFs. Of these six, one is part of the eight primary studies considered relevant for this umbrella review [50]. This same conclusion, based only on the results of Mangano et al., (2018) [50], was also given by Werny et al., (2022) [42] and Alsulaimani et al., (2022) [41].

Bazal-Bonelli et al., (2022) [43] observed that the patients included in one of the studies reviewed [70], presenting sufficient serum vitamin D levels, were found to undergo significantly less bone loss measured at one year compared with deficient serum vitamin D levels. However, the SR authors also observed that, according to Mangano et al., (2018) [50], no significant differences were found between survival of dental implants in patients with deficient vitamin D serum levels and in patients with sufficient serum levels of vitamin D. This same conclusion, related to the Mangano et al., (2018) [50] results, was also given by Makke (2022) [40]. Although the Tabrizi et al., (2022) [70] study is more recent and has good methodological quality (6/9 according to the Newcastle-Ottawa scale), Bazal-Bonelli et al., (2022) [43] gave more weight to the results of the Mangano et al., (2018) [50] study because it has a larger sample size (885 vs. 90 patients) and a much longer follow-up (168 vs. 12 months) compared to the Tabrizi et al., (2022) [70] study.

Although these criteria have a significant impact on the methodological quality of the studies, it was considered that this was not a sufficient justification to discard the results, and, therefore, an analysis of the Tabrizi et al., (2022) [70] study was carried out. According to these authors, the mean marginal bone loss (MBL) was higher in patients with deficient serum vitamin D levels (1.38 ± 0.33 mm; 8.13 ± 0.78 ng/mL) when compared to patients with sufficient vitamin D (0.78 ± 0.12 mm; 35.37 ± 3.66 ng/mL). For 50.6% of patients, the mean marginal bone loss could be accounted for by the low serum vitamin D level (R^2^ = 0.506, *p* = 0.001, 95% confidence level), and for every one-unit increase in serum vitamin D level, the mean MBL decreased by 0.02 mm (*p* < 0.001, 95% confidence level). However, the difference in mean MBL between patients with sufficient and deficient serum levels of vitamin D was 0.60 mm at the 1-year follow-up, which may not be clinically significant.

It is possible to observe that different authors ([39,41,42] vs. [40,43]) drew different conclusions regarding the analysis of the Mangano et al., (2018) [50] study, with some arguing that there is a positive association between vitamin D levels and the success of dental implants and others arguing that this association does not exist, respectively. Therefore, it was felt necessary to specifically analyze this study, and it was concluded that a tendency for EDIF to increase in patients with vitamin D deficiency was observed, although the difference was not statistically significant (*p* = 0.105). Ultimately, this means that none of the authors of these five systematic reviews are completely right. The conclusion drawn from the Mangano et al., (2018) [50] study is that, although there is a positive association between low levels of vitamin D and EDIF, the results are not statistically significant. Still, we note that such a statement does not mean that the null hypothesis true.

### 6.2. Optimal Level of Vitamin D and Success of Dental Implants

The results of Hakim et al., (2021) [39] showed some evidence of the association between vitamin D levels and the success rate in dental implantation. Of the five studies that reached these conclusions, two are part of the eight primary studies considered relevant for this umbrella review [47,49].

Alsulaimani et al., (2022) [41] state that it is difficult to find a direct relation or causality between the low serum vitamin D level and EDIF and that vitamin D may play a role in dental implant success through its effects in modulating the immune system and healing process; however, it was based on only three included studies [48,50,68]. In the remaining discussion, the authors analyzed studies that were not included in the selection phase and took into account their results in the conclusions of the review. By contrast, they did not discuss the results of the remaining three included studies, all being part of the eight relevant primary studies [8,49,51]. Taking this information into account and through individual analysis of the four relevant primary studies included, it is possible to suggest that implant placement can be successful after vitamin D supplementation [49], that vitamin D supplementation has beneficial effects on bone turnover and regeneration in patients with a deficit [8], and that there is a clear association among vitamin D deficit, reduced osseointegration, and increased EDIF incidence in both animal and human studies [51]. Contrary to what the SR authors reported, it should have been mentioned that the other two studies did not obtain statistically significant results [48,50].

Werny et al., (2022) [42] state that the supplementation of vitamin D appears to enhance the osseointegration in animals with systemic diseases and that only slight evidence supports the hypothesis that humans similarly benefit from it. However, deleting information regarding animals with pathology and other studies with eligibility criteria that do not agree with those of this umbrella review, the literature suggests that local vitamin D supplementation enhances the BIC [47] but also that systemic vitamin D supplementation reduces symptoms associated with vitamin D deficiency, helps patients with disturbed mineral homeostasis [49], and enhances new bone formation around dental implants in humans [53].

According to Bazal-Bonelli et al., (2022) [43], patients supplemented with vitamin D underwent less bone reduction than patients who were not supplemented. Of the three studies that led the authors to this conclusion [52,53,70], two of them [52,53] are part of the relevant primary studies.

The findings of Makke, (2022) [40] are contradictory since, taking into account only the relevant primary studies, the author found one human study with favorable results [49] and two human studies that found no significant relationship between vitamin D and dental implants [48,50].

### 6.3. Supplemented Doses of Vitamin D

None of the SRs, which included experimental or case-report studies, mentioned the supplemented dose of vitamin D. Analyzing each of the primary studies individually, of the four that were supposed to mention these data [47,49,52,53], only both RCTs mentioned it [52,53]. In Garg P et al., (2020)’s study [36], the EG were supplemented with a cholecalciferol sachet of 60,000 IU/month for three months and continued for six months depending on the level of vitamin D. In Kwiatek et al., (2021)’s study [53], the patients were instructed to take 8000 IU/daily for 12 weeks. It is clearly evident that the dosage differs greatly from one study to another; however, the results of both studies are concordant and conclude that vitamin D supplementation is an effective approach to improving the osseointegration of dental implants.

### 6.4. Vitamin D Theories

In addition to the direct influence of vitamin D on the success or failure of dental implants, the included SRs also presented some theories that may justify the relationship between these two factors.

According to Hakim et al., (2021) [39] and Makke (2022) [40], the role of vitamin D in the calcium economy is extremely important. During osseointegration, calcitriol affects the processes of activation and differentiation of osteoblasts and osteoclasts. This theory is also supported by Pereira et al., (2019) [56], Satué et al., (2016) [71], Acipinar et al., (2019) [72], Bhandage et al., (2022) [73] and Singh et al., (2023) [74]. 

Vitamin D has also been found to be essential for the maturation and proper functioning of bone cells for increasing osteoid mineralization [71] that can play an important role in the stabilization phase of the implant, seems to have potent anti-inflammatory effects, and may be essential in modulating the innate and adaptive immune responses and for the antibacterial response [39,43]. There was a significant correlation between infection rate and vitamin D levels of < 20 ng/mL. This theory is also supported by Acipinar et al., (2019) [72] and Diachkova et al., (2021) [75].

Therefore, the optimal vitamin D level might be one of the most critical systemic factors in preventing infection and providing an optimal environment for successful osseointegration (Makke 2022) [40].

Moreover, it has been observed that DIFs decrease threefold when dental implants are placed by digitally guided surgery compared with freehand placement (2.25% vs. 6.42%) (Bazal-Bonelli et al., 2022) [43].

Still, the authors of the systematic reviews could have delved even deeper into the possible reasons that justify the influence of the vitamin on the success of dental implants.

## 7. Discussion

An umbrella review such as this one has the main objective of analyzing and summarizing in the best possible way all the existing information in SRs on a specific topic, considering certain eligibility criteria. In this way, it allows to present the state-of-the-art of this same subject, up to the present time, to assist decision-making in clinical practice and to know the next steps that must be taken in terms of scientific research.

This umbrella review brought together five SRs, which, in turn, allowed the analysis and presentation of results from eight primary studies, although, in all, they gathered 29 different studies. This was due to the fact that the SRs did not elaborate specific eligibility criteria and to errors in the study selection process.

This umbrella review did not include grey literature, which may represent publication bias. Nevertheless, we consider that the databases used to select the studies are very comprehensive and, therefore, increase the probability that all relevant studies were included.

Generally, an umbrella review does not analyze the primary studies included by SRs. However, in the present study, since numerous limitations were identified in the included SRs, the need to individually analyze the studies included by them was felt. In this individual analysis of the 29 primary studies, it was noticed that the majority did not respond to the main problem of this umbrella review due to the various reasons already mentioned in the results.

Despite this major limitation, the analysis of the eight primary studies carried out by the five SRs and by the present umbrella review is sufficient to present the state-of-the-art of the subject up to the present time, to identify the main limitations of the study of the subject, and to guide future researchers.

In methodological terms, several flaws were found in the SRs that, most likely, called their quality and internal validity into question and what, in the future, should be avoided. For instance, in only one of the SRs [43], it is mentioned that the literature search and/or data extraction were carried out by two researchers independently, as it should be performed. Since this is one of the points evaluated on Joanna Briggs’ scale, this is one of the reasons why the remaining SRs do not score as high. This flaw makes the study selection process less reliable, increasing the likelihood of selection errors. This may be the reason why some SRs included studies where implants were placed in other limbs or studies where vitamin D was not even evaluated. In this way, knowing that the selection process of the studies of the five SRs was not carried out correctly, this umbrella review does not guarantee that it brings together all existing studies in the literature, in Portuguese, English, or Spanish, that evaluate the effect of vitamin D on the success of dental implants.

This same SR [43] was also the only one that mentioned having evaluated the methodological quality of the primary studies. As the others do not mention such information, it must be assumed that they did not carry out this phase of investigation. This is another of the points evaluated on the Joanna Briggs scale, which also compromised the final score of the methodological quality of the remaining SRs and is essential to understand how the results of primary studies may be negatively influenced by errors in the studies themselves.

Human studies were performed as cross-sectional, case-report, and RCT. These variations on the study design make it difficult to come to a solid conclusion on the subject. As mentioned before, the most reliable studies after SRs and MA studies are RCTs because they can guide scientists accurately to resolve scientific gaps. Case reports can help the researchers in cases of a lack of good sample size, and cross-sectional studies are performed to study deceptive aspects of a population at a specific time; however, they cannot determine the relation or cause of something. The retrospective studies have only the possibility of analyzing the available information and data, with no possibility of manipulating the variables in the present [76].

Other limitations observed are related to the lack of characterization of the sample and of the EG and CG. This information was not established in the eligibility criteria nor described in the results of the SR, resulting in heterogeneous samples and a greater probability of the results being non-significant. This heterogeneity also made it difficult to compare the results of the different SRs since different populations with different results cannot be fairly compared.

An inconsistency in the reference values of vitamin D was also observed, impairing the reliability of comparisons made between studies. In addition, none of the SRs, which included experimental or case-report studies, mentioned the supplemented dose of vitamin D, indicating the presence of an information bias. Analyzing each of the primary studies individually, of the four that were supposed to mention these data, only both RCTs did [52,53]. This is a major failure of the SR, since the lack of this information does not allow a fair comparison to be made between studies and makes it impossible for their methodology to be reproducible by future researchers.

Furthermore, the only two studies that indicated the dosage of supplementation present completely different dosages. In one study, the patients were supplemented with cholecalciferol sachet 60,000 IU/month [52]—which is equivalent to approximately 2000 IU/day—and in the other, the supplementation was 8000 IU/day [53]—which is equivalent to approximately 240,000 IU/month. Although this decision was not explained by the authors, it is possible that the duration of treatment influenced the decision on dosage. Although the dosage in the first study appears to be significantly lower than that in the second one, the multiplication of the dosage by the duration of treatment shows, overall, that the patients in the Garg et al., (2020) study [52] took 540,000 IU and the patients in the Kwiatek et al., (2021) [53] study took 672,000 IU. Although the difference between both studies is still large, it is not as disproportionate as it initially seemed. Nevertheless, it is a factor that undoubtedly influenced and contributed to the difference in results, despite both demonstrating a positive association between vitamin D levels and dental implant success. If we look at Figure 2, which shows the recommendations for treatment of vitamin D deficiency in adults, it can be seen that, according to the USA Endocrine Society (2011), the treatment for the general population must consist of taking 6000 IU/day for eight weeks, and, according to the Central European EVIDAS (2013), the treatment must consist of taking 7000–10,000 IU/day for 4 to 12 weeks. Therefore, of the two RCTs analyzed, the one that comes closest to the recommendations is the one in the Kwiatek et al., (2021) [53] study. To prevent this discrepancy from occurring again, it is suggested that, in future studies, the dosage of vitamin D supplementation, in addition to always being specified, should follow the recommendations of the country or continent in question so that it can be standardized. This will allow future umbrella reviews to compare the results of different studies more fairly and clearly.

Although the main objective of all studies was to evaluate the effect that optimal levels of vitamin D have on dental implants, compared to low levels, some differences in the evaluation method were observed. Some looked at it through vitamin D supplementation, and the others assessed how low levels of this hormone influence EDIF.

Since five of the eight primary studies were included in more than one SR, an analysis and comparison of their conclusions was carried out in order to verify if there is any inconsistency or flaw in the presentation of the results. According to this analysis, three differences were identified. Werny et al., (2022) [42], when presenting Mangano et al., (2018)’s results [50], only mention that a higher prevalence of EDIF was observed in patients with lower vitamin D levels, hiding that these results were not statistically significant. This same author, as well as Hakim et al., (2021) [39], when analyzing the results of Salomó-Coll et al., (2016) [47], mention only the positive results, hiding the variables where the optimal levels of vitamin D did not prove to be significantly effective. This indicates the presence of a confirmation bias, that is, the tendency to seek out and prefer information to bolster a hypothesis, resulting in the tendency to ignore any information that contradicts those beliefs. This bias, just like in the others, could have been unintentional but can still lead to poor decision-making since it does not report the true result. Although the objective of most authors, when carrying out research studies, is to demonstrate the effectiveness of a variable, it is important that its development is as impartial as possible and that all results are presented, even when they are not favorable to research, in order to provide reliable data that can be extrapolated to clinical practice.

While there appears to be a positive association between vitamin D levels and the success of dental implants, there are naturally many other factors that may influence this process, such as age, diet, smoking, alcohol consumption or other drugs, stress, and daily oral hygiene, among others [77]. Given that the main objective of these systematic reviews and, consequently, of this umbrella review was to analyze the influence of vitamin D levels, it would be expected that all other factors would not receive as much attention and weight in the results. Therefore, it can be hypothesized that the inconsistency of some results is due to the influence of internal or external factors other than vitamin D levels. Unfortunately, this hypothesis could not be tested in this umbrella review since none of the analyzed studies took these factors into account, with the exception of age [41].

Therefore, it is considered essential that future primary studies, even with the same study objective, take into account factors other than vitamin D, that they can control these factors (either by exclusion/inclusion or by dividing groups), and that they take them into account in the analysis of results and final conclusions.

In addition to the previously mentioned limitations, another one detected was related to the *p*-value. It is known that the threshold of statistical significance that is commonly used is a *p*-value of 0.05 and that the smaller the *p*-value, the greater the statistical incompatibility of the data with the null hypothesis. However, the *p*-value depends on other variables, and its statistical significance does not automatically equate to scientific, human, or economic significance, so a low or a high *p*-value is not the sole basis for a scientific claim [78]. In most of the SRs included, an abuse was observed in the way the *p*-value was evaluated, since all results with a *p*-value greater than 0.05 were considered to be of “no importance” or “valueless”, ignoring the remaining factors such as inappropriate study design, imprecise measurement, erroneous statistical analysis, or small sample size. *p* > 0.05 only means “no evidence of difference”, which does not mean “no difference between the groups” [79]. However, this abuse was not observed in the analysis of any of the primary studies considered relevant for this review.

Even so, when we say that, for example, Mangano et al., (2016) [48] and Mangano et al., (2018) [50] did not obtain statistically significant results, with a value of *p* = 0.15 and *p* = 0.105, respectively, despite it not being an abusive conclusion, it is important to note that this does not mean that vitamin D does not influence, in any way, the osseointegration of dental implants. Although the results are not always statistically significant, the general trend of the results towards a positive relationship between vitamin D levels and the success of dental implants is clear, and this trend has great clinical relevance.

Despite the methodological differences between the SRs, consistency was found in most of their conclusions.

It is difficult to find a direct relation or causality between vitamin D and implant failure based on the current research in the literature. The studies’ findings were inconsistent, but some of this research showed a clear association between vitamin D and osseointegration, marginal bone loss, dental implant survival, and EDIF [39,40,43].

The supplementation of vitamin D appears to enhance the osseointegration in animals with vitamin D deficiency, although only slight evidence supports the hypothesis that humans similarly benefit from vitamin D supplementation in terms of osseointegration [42]. Although this evidence may be superior in animals, this is how clinical research develops, and, according to the data collected, we can conclude that research on the subject will increase and improve its quality in humans.

Since none of the existing systematic reviews had yet demonstrated homogeneity of results and a clear and positive support for the influence of vitamin D on osseointegration in humans, it was considered advantageous to include studies carried out in animals since their positive results could be an added value to justify the need to develop further studies in humans in the hope that the results would be comparable and reproducible. Even so, since the results of studies carried out in animals can never be extrapolated to clinical practice in humans, the results presented here from preclinical studies did not influence in any way the conclusions given on clinical studies.

Vitamin D may play an important role on all the variables mentioned through its effects in modulating the immune system modulation and healing process [39,41]. The physiological processes that explain and justify the influence of vitamin D on osseointegration of dental implants are still unclear, but all theories mentioned by the SR are valid and should be tested in future studies.

Additional large-scale human prospective clinical studies and case reports are required to determine the association between serum vitamin D level and osseointegration and to approve the hypothesis that vitamin D may play an important role in improving implant success through its effect on the immune system modulation.

In patients with severely compromised vitamin D levels, the recommendation is to supplement with vitamin D to ensure optimal treatment outcomes.

## 8. Conclusions

The development of this umbrella review allows us to conclude that the publication of studies on the effect of vitamin D on the osseointegration of dental implants has been increasing much in recent years. The evolution found in these studies is clear, moving from pre-clinical to clinical studies with ever greater methodological quality.

According to the available literature and data analyzed in this umbrella review, it is possible to state that vitamin D may have a positive effect and play an important role on the osseointegration, in reducing bone loss, dental implant survival, and consequently, on the reduction of EDIF, that is, on the success of dental implants.

However, there is still some inconsistency in the results, and the study of this topic still has much to evolve.

Furthermore, it is important to take into serious consideration that vitamin D levels are not the only factor that influences the osseointegration process and that contributes positively to the success of dental implants and, therefore, should be seen as a contributing factor and not as the sole determinant of dental implant success.

As mentioned by the authors of the included SRs and given the high percentage of patients with vitamin D deficiency, it may be advisable to determine the serum vitamin D level of each patient before placing dental implants. The development of further higher-level studies, such as prospective clinical research studies and mainly RCTs, to investigate the correlation or causal relationship between serum levels of vitamin D and risk of EDIF is also recommended. It is imperative to develop studies with this study design with larger sample sizes and different follow-up periods.

In future studies, to complement the disadvantages of *p*-values, some statisticians recommend the supplementation or replacement of *p*-values with other statistical methods, including confidence, credibility or prediction intervals, likelihood ratios, Bayesian statistics, and decision-theoretic modeling, focusing more on estimation than testing.

In the future development of SRs, it is recommended that each of the phases is carried out by more than one researcher and that decisions are discussed between them to ensure that all existing information is selected and analyzed and, consequently, made available to the best scientific evidence.

## Figures and Tables

**Table 1:** Characteristics of the included SRs.

Author, Year	Aims	Search Strategy	SR Included/ Sample	Risk of Bias	Authors’ Conclusions	JBI Score
[39]	To evaluate the association between vitamin D and EDIF.	PubMed, Scopus, Web of Science, Cochrane, and EMBASE. Study selection was performed by two authors independently. It is not mentioned by how many researchers the data extraction is performed. Referred keywords. Assessment of methodological quality is not mentioned.	Nine human studies (cross-sectionals, case reports, case-controls, and one RCT) and three animal studies.	----	Contradictory results were found regarding the relationship between vitamin D and the success of dental implants. However, the authors suggest that vitamin D may play a role in improving implant success through its effects on immune system modulation.	6/11
[40]	To investigate the available literature regarding vitamin D supplementation and dental implant osseointegration.	PubMed, Scopus, ResearchGate, and Google Scholar. It is not mentioned by how many researchers the study selection and data extraction is performed. Referred keywords. Assessment of methodological quality is not mentioned.	Five human studies (retrospective, case series, and case reports) and six animal studies.	----	A significant relationship between vitamin D and osseointegration was found, demonstrating the importance of this supplementation in the short- and long-term outcomes after dental implant treatment.	4/11
[41]	To investigate the relationship between low serum levels of vitamin D and EDIF and determine the amount of vitamin D that can affect the implant survival rate.	PubMed, Directory of Open Access Journals, and Web of Science. It is not mentioned by how many researchers the study selection and data extraction is performed. Referred keywords. Assessment of methodological quality is not mentioned.	Six human studies (case-controls, case reports, and reviews).	----	Despite the high success of dental implants, FDI has also been observed. However, the authors support the role of vitamin D in bone mineralization and maturation, immunity, and inflammatory responses.	8/11
[42]	To review the available evidence to evaluate the efficacy of vitamin D supplementation or vitamin D depletion on the osseointegration of implants in animals and humans.	PubMed/Medline, Cochrane Library, and Google Scholar. Study selection was performed by one researcher, but it is not mentioned by how many researchers data extraction is performed. Referred keywords. Assessment of methodological quality is not mentioned.	Five human studies (case-controls, case reports, and reviews) and 13 animal studies.	----	Vitamin D deficiency seems to have a negative effect on the osseointegration of implants in animals, and the supplementation appears to enhance it in animals with vitamin D. However, it does not appear clear evidence that supports the hypothesis that humans similarly benefit from vitamin D supplementation in terms of osseointegration.	7/11
[43]	To investigate the relationship between serum vitamin D levels and dental implants in terms of survival rates, marginal bone loss, and associated complications.	PubMed/Medline, Cochrane Library, Scopus, and Web of Science. Study selection and data extraction was performed by two researchers independently. Referred keywords. The Cochrane Handbook of Systematic Reviews and Interventions was used to assess the risk of bias of RCT, and the Newcastle-Ottawa scale was used to assess the quality of others.	Four human studies (retrospective cross-sectional and RCT).	Clear assessment of the methodological quality of primary studies.	It appears that serum vitamin D levels in patients may play a relevant role in osseointegration, marginal bone loss, and dental implant survival. In this way, it is advisable to determine the vitamin D level of each patient before placing dental implants and to provide vitamin D supplementation when necessary.	9/11

**Table 2:** Characteristics of the excluded studies.

Author, Year	Exclusion Criteria
[44]	The location of the implants does not correspond to the intended location in this study.
[45]	The SR does not answer the question of the present umbrella review.
[46]	It is a narrative review and not a SR.

**Table 3:** Methodological quality of the included SRs.

Authors/ Items	[39]	[40]	[41]	[42]	[43]	TY
1	N	N	Y	Y	Y	3 (60%)
2	Y	N	Y	Y	Y	4 (80%)
3	Y	N	Y	Y	Y	4 (80%)
4	Y	Y	Y	Y	Y	5 (100%)
5	U	N	Y	N	Y	2 (40%)
6	U	N	N	N	N	0 (0%)
7	N	N	N	N	Y	1 (20%)
8	Y	Y	Y	Y	Y	5 (100%)
9	NA	NA	NA	NA	NA	NA
10	Y	Y	Y	Y	Y	5 (100%)
11	Y	Y	Y	Y	Y	5 (100%)
**TY**	6 (54.5%)	4 (36.4%)	8 (72.7%)	7 (63.6%)	9 (81.8%)	

**Table 4:** Characteristics of the studies included in the five SRs selected by this umbrella review’s eligibility criteria.

Author, Year	Aims	Study Design	Sample Size	Authors’ Main Conclusions
[8]	Summarize the state-of-the-art biological risk factors in bone grafting in implantology.	Narrative review	---	The vitamin D serum level plays a predominant role in bone metabolism since it stimulates the activity of osteoclasts and increases the production of extracellular matrix proteins by osteoblasts.
[47]	To evaluate the effect of topical application of Vitamin D over implant surface throughout analysis of peri-implant tissue.	Animal study	Six dogs TG: 12 implants supplemented with 10% of Vitamin D CG: no treatment	Topical application of vitamin D during immediate implant treatment seems to not have an enhanced effect on dental implant osseointegration, although the supplemented implants exhibited less crestal bone loss and 10% more BIC.
[48]	To investigate whether there is a correlation between EDIF and low serum levels of vitamin D.	Retrospective cross-sectional study	822 patients and 1625 implants	Although the incidence of early implant failures was higher in patients with low serum levels of vitamin D, the study failed in proving an effective link between both variables.
[49]	Illustrate two patients with vitamin D deficiency and EDIF and demonstrate that implant placement was successful after supplementation.	Case-report	Two patients	Implant placement was successful after vitamin D supplementation in patients with vitamin D deficiency and EDIF.
[50]	To investigate whether there is a relationship between low serum levels of vitamin D and EDIF.	Retrospective cross-sectional study	885 patients and 1740 implants	No statistically significant correlation was found; however, a clear trend toward an increased incidence of EDIF with lowering of serum vitamin D levels was reported.
[51]	Summarize the role of dietary supplements in optimizing osseointegration after implant insertion surgery.	Scoping review	19 articles included	There is a clear association among vitamin D deficit, reduced osseointegration, and increased EDIF incidence in both animal and human studies.
[52]	To evaluate the crestal bone level in patients having low levels of vitamin D treated with dental implants with or without vitamin D3 supplements.	RCT	32 patients EG: VD3 supplements CG: no treatment	Cholecalciferol has systemic effects on accelerating bone formation around titanium implants.
[53]	To assess what effect the 25-hydroxycholecalciferol concentration and vitamin D deficiency treatment have on changes in the bone level at the implant site during the process of osseointegration in the mandible.	RCT	122 patients GA: low level of vitamin D and no treatment GB: low level of vitamin D and supplementation GC: normal level of vitamin D.	The correct level of 25-hydroxycholecalciferol on the day of surgery and vitamin D deficiency treatment have a significant influence on the increase in bone level at the implant site during the process of osseointegration.

## Data Availability

Not applicable.

## Abbreviations

BIC	Bone-to-implant contact
EDIF	Early dental implant failure
JBI	Joanna Brigs Institute
MA	Meta-analysis
PRISMA	Preferred Reporting Items for Systematic Reviews and Meta-Analyses
RCT	Randomized controlled trial
SR	Systematic review
